# Impact of the COVID-19 Pandemic on Paediatric Dental Treatment: A Retrospective Study in Banja Luka, Bosnia and Herzegovina

**DOI:** 10.3390/ijerph191912292

**Published:** 2022-09-27

**Authors:** Olivera Dolic, Marija Obradovic, Zeljka Kojic, Natasa Knezevic, Natasa Trtic, Valentina Veselinovic, Slava Sukara

**Affiliations:** Department of Dentistry, Faculty of Medicine, University of Banja Luka, 78000 Banja Luka, Bosnia and Herzegovina

**Keywords:** COVID-19, SARS-CoV-2, pandemic, dentistry, paediatric patients, public health, paediatric dental treatment

## Abstract

Dentists are at significant risk of COVID-19 infection. It was difficult to find a balance between dental care, especially preventive and other non-urgent dental procedures, and prevention of potential exposure to SARS-CoV-2 infection. The aim of the present study was to assess the influence of the COVID-19 pandemic on dental treatment in children in the Dental Clinic of the University of Banja Luka, and to compare it before and during the first and second years of the pandemic. All dental records of paediatric patients who attended the Dental Clinic (for the period March 2019 to March 2022) were analysed. The data on selected dental treatment procedures were divided into three groups per year and compared. The results during the first year of the COVID-19 pandemic showed a reduction in single treatments compared to the year before, while in the second year there was an increase in some interventions such as oral hygiene training and patient motivation, deciduous tooth extraction, and glass ionomer filling. Although the number of dental treatments in the clinic in the second year nearly returned to pre-pandemic levels, preventive and restorative interventions are the most appropriate strategy to improve the oral health of children after the COVID-19 pandemic.

## 1. Introduction

In recent decades, emerging infectious diseases have increased worldwide. The first cases of atypical pneumonia of unknown cause were recorded in the city of Wuhan in the Chinese province of Hubei and recognised as a novel coronavirus, designated as 2019-nCoV. Flügge droplets (size greater than 5 microns), that are emitted when speaking, coughing, or sneezing [1], and Wells droplets (diameter less than 0.1 microns), with less possibility of transmission from micro-organisms, are the main routes of transmission of COVID-19. In addition, it is possible to transmit the virus by aerosol if the concentration of the virus is rather high in a contained space, and most dental procedures are aerosol-generating procedures [2]. A patient, with his or her mouth open while wearing no mask and maintaining no necessary physical distance, represents a two-way risk of contracting the infection, in either a direct or indirect manner. This risk was more serious in paediatric dentistry because affected children frequently present asymptomatic, mild, or moderate clinical manifestations [3]. To reduce the spread of infection between dentists, patients, and other healthcare providers, the World Health Organisation (WHO) has updated and added additional safety measures and protocols before and during dental treatment [4].

The WHO declared the pandemic of SARS-CoV-2 or COVID-19 disease on 11 March 2020 [5]. The first patient in Banja Luka, Bosnia and Herzegovina, was diagnosed on 5 March 2020. Schools, colleges, kindergartens, as well as the work of dental clinics, except for three public dental institutions in the city of Banja Luka, stopped their activities on 11 March 2020. In the first months of the pandemic, the Dental Clinic of the Faculty of Medicine of the University of Banja Luka was one of three dental institutions in Banja Luka that limited their activities to nondeferrable emergency care, as did many dental clinics worldwide. After that period, all elective dental treatment procedures were performed for all patients with no suspicion or symptoms of COVID-19 infection. 

Several studies have investigated the impact of the COVID-19 pandemic on dental practice [6,7,8,9,10,11,12,13,14]. In some of the countries included in these studies there were several lockdowns [14], and a large percentage of the population, including children, was vaccinated after the introduction of the vaccine. However, in Bosnia and Herzegovina, the percentage of the vaccinated population is small (36.48%) [15]. Vaccinating children older than 5 years is allowed in Bosnia and Herzegovina, but the number of vaccinated children is negligible, and no information could be obtained on the prevalence of vaccinated children. Although paediatric dentistry is essential in children’s healthcare, it was difficult to find a balance between dental care, especially preventive and other non-urgent dental procedures, and prevention of potential exposure to SARS-CoV-2 infection [16,17,18]. The aim of the present study was to assess the influence of the COVID-19 pandemic on dental treatment in children at the Dental Clinic of the Faculty of Medicine of the University of Banja Luka and to compare it before and during the first and second years of the pandemic. 

## 2. Materials and Methods

This retrospective cross-sectional study was approved by the Ethical Committee for Research Conducted on Humans and Biological Material of the Faculty of Medicine, University of Banja Luka (no. 18/4.78/22; 07/03/2022). All dental records (977 records) of paediatric patients who attended the Dental Clinic of the University of Banjaluka between March 2019 and March 2022 were included. 

The data on selected dental treatment procedures in preventive and prophylactic dentistry, extractions, conservative dentistry, and endodontics were divided into three groups—dental treatment procedures before the pandemic (11 March 2019–10 March 2020), dental treatment procedures in the first year of the pandemic (11 March 2020–10 March 2021), and dental treatment procedures in the second year of the pandemic (11 March 2021–10 March 2022)—and compared. The selective procedures in preventive and prophylactic dentistry were oral hygiene training and patient motivation, fluoride varnish, fissure sealant, and dental plaque removal. The data on conservative procedures included glass ionomer filling, composite filling on anterior teeth, composite filling on posterior teeth, and temporary filling. The temporary endodontic procedures and root canal filling were selective endodontic procedures. The data were collected by one qualified dentist through the electronic filing system. 

### Statistical Analysis

The data were arranged into three groups in a Microsoft Excel spreadsheet and imported into SPSS V.25.0 (IBM Corporation, Armonk, NY, USA) for statistical analysis. A descriptive analysis reported the frequencies and percentages of the outcomes. A chi-square test was used to analyse differences among participants with regard to the total number of new registered paediatric patients and gender. In addition, the chi-square test was used to compare and Pearson’s test was used to correlate the dental procedures before and during the first and second years of the pandemic period. The level of significance was set at *p* < 0.05.

## 3. Results

Table 1 shows the total number of newly registered paediatric patients before, during the first year of, and during the second year of the pandemic. The number of dental treatments before the COVID-19 pandemic and during its second year was similar, with 2757 and 2585 cases, respectively. In the first year of the COVID-19 pandemic, the number of dental procedures was reduced by 40% compared to the year before the COVID-19 pandemic and amounted to 1652 cases. We found statistical significance (χ^2^ = 73.1, *p* < 0.01) in comparing the number of new patients registered in each year studied. No statistical significance was found regarding gender. 

**Figure 1:**
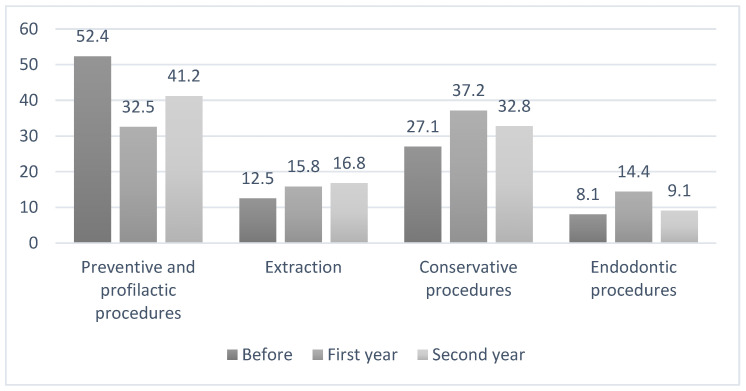
The percentages of dental procedures (grouped by similarity) before, during the first year of, and during the second year of the COVID-19 pandemic.

Figure 1 shows the percentages of dental procedures before and during the first and second years of the COVID-19 pandemic. The preventive and prophylactic measures in the year before the pandemic amounted to 1444 (52.4%) cases, but in the first and second years, we can see a drop provision of these procedures, with 538 (32.5%) and 1066 (41.2%) cases, respectively. In the first year of the pandemic, the most common procedure was restorative treatment, with 614 (37.2%) cases. The least common procedure in all years was endodontic treatment, with 222 (8.1%), 238 (14.4%), and 236 (9.1%) cases, respectively.

Table 2 presents the comparisons and their significance regarding the procedures before and during the first year of the COVID-19 pandemic. The highly statistically significant procedures in these two periods were fluoride varnish, dental plaque removal, deciduous tooth extraction, glass ionomer filling, composite filling on posterior teeth, temporary filling, temporary endodontic procedures, and root canal filling. Moreover, Pearson’s correlation confirmed the significant positive correlations in procedures between the two periods (r2 = 0.6; *p* = 0.03).

Table 3 presents the comparisons and their significance regarding the procedures before and during the second year of the COVID-19 pandemic. Similar to the comparison in Table 1, the highly statistically significant procedures in these two periods were fluoride varnish, dental plaque removal, deciduous tooth extraction, glass ionomer filling, temporary filling, temporary endodontic procedures, and root canal filling. The difference compared to the results from Table 1 is that the statistical significance in these results existed for oral hygiene training and patient motivation and composite filling on anterior teeth. Moreover, Pearson’s correlation confirmed the significant positive correlations in procedures between the two periods (r2 = 0.8; *p* < 0.01).

Table 4 presents the comparisons and their significance regarding the procedures during the second year of the COVID-19 pandemic relative to the first year of the COVID-19 pandemic period. Fluoride varnish and temporary filling were procedures with high statistical significance. Moreover, Pearson’s correlation confirmed the significant positive correlations in procedures between the two periods (R = 0.9; *p* < 0.01).

## 4. Discussion

Summarizing the results of the impact of the pandemic on the provision of dental services in children, we should emphasize a significant drop in preventive and prophylactic measures during the first year of the pandemic (32,57%). This percentage increased in the second year (41.2%); however, it remained significantly lower compared to the period before the beginning of the emergency situation (52.4%). Dental care for children in Bosnia and Herzegovina is primarily provided in public health institutions and is free for children under the age of 15. It is provided to a lesser extent in private dental institutions. According to a recent survey by Vuković et al., 92 paediatric dentists were registered in Bosnia and Herzegovina representing only 4.1% of the total number of registered dentists (2250) [19]. In addition, dental disease prevention in Bosnia and Herzegovina is poor, there is no national prevention plan or protocols that is consistently implemented. High caries prevalence among our children, especially those of low socio-economic status or lack of access to dental care, is an indicator [20,21]. Unfortunately, with a reduction in the provision of preventive measures, COVID-19 worsened an already unfavourable situation.

The total number of new paediatric dental patients in the year before COVID-19 was 242. Results showed a drop in newly registered paediatric dental patients during the first year of the pandemic (181) and an increase in new admissions during the second year (337).

Alamoudi et al. also recorded that the number of paediatric dental patients decreased by 60.7% in the period from March 2020 to December 2020 during COVID-19 compared to the same period in the previous year (March 2019–December 2019) [11]. The survey conducted in San Paolo Hospital in Milan, divided into three age groups—children (≤18 years), adults (≥19 to ≤65 years), and elderly (>65 years)—included data of 901 admissions and recorded a decrease in admissions during the lockdown and the second wave periods of −67.37% and −40.35%, respectively, when compared to the pre-COVID period [12]. On the other hand, Eggmann et al. found an increase in demand for urgent dental care during the lockdown. They state that the daily patient volume in the emergency service was, on average, 32.9 in the pre-lockdown period, 41.5 in the lockdown period, and 40.8 in the post-lockdown period [22].

A reduction in single treatments compared to the year before was observed during the first year of COVID-19, while in the second year we saw an increase in some interventions such as oral hygiene training and patient motivation, deciduous tooth extraction, and glass ionomer filling. As already mentioned, in Bosnia and Herzegovina a state of emergency was declared in March 2021. A lockdown was implemented, and citizen movements were restricted. All non-urgent dental interventions stopped, and only emergency dental treatments were provided until the state of emergency was lifted (late May 2021) [23]. Ultrasonic scaling, tooth restoration, and endodontic access cavity with rotary tools, as well as surgical extraction with rotary tools were listed among the high-risk procedures [4]. Accordingly, in our dental paediatric clinic, we used atraumatic restorative treatment and other minimally invasive procedures and avoided using aerosol generating procedures when providing dental treatment during the first two months of COVID-19. Before entering the clinic, the probability of SARS-CoV-2 infection was assessed for each patient using a questionnaire. They were asked if they had any symptom consistent with COVID-19, if they had close contact with a patient who tested positive for COVID-19, and if they had travelled to an area of high risk for COVID-19. In addition, their temperature was measured at the triage station at the clinic’s entrance. After a positive response or elevated body temperature, the patient was not treated in our clinic because a special dental unit for patients with suspected or positive COVID-19 was established at the level of public health dental protection. All other patients were treated using infection prevention measures [4]. 

After the lockdown period, as of the beginning of June 2019 until now, questions about suspicion or symptoms of COVID-19, as well as temperature measurement (of suspected cases) before entering the dental clinic remain. All dental procedures have been provided in the same way as before COVID-19 but with particular caution, such as the use of additional protective equipment such as an N95 mask and face shield. Face shield use is not mandatory during periods when the epidemiological situation is more favourable. Although many patients had been waiting for dental services to open again, the COVID-19 safety protocols certainly reduced the number of patients and interventions. In the first months, requests for individual dental treatments were mainly related to emergency interventions. Despite the fact that we started providing almost all of our dental services, requests for certain treatments were reduced. We consider that the fear factor influenced many to avoid coming to the dentist. This premise has been confirmed in research by Ibrahim et al., who delineated that higher fear to seek dental care was significantly associated with higher fear of COVID-19 and perception of higher risk of infection in dental settings [24].

Regarding single dental treatment, in the year before the pandemic, the most frequently performed procedure was preventive fluoride varnish application (21.04%), while during the first and second years of the pandemic, it was glass ionomer filling (21.43% and 19.73%, respectively). A drop in preventive procedures occurred in the first year, and this trend was mostly continued during the second year, except for oral hygiene training and patient motivation. In their study in Israel, Elalouf et al. showed that sealant, space maintainer, and tooth fixation were not practiced at all in paediatric patients during the lockdown [25]. Samuel et al. also found a significant drop in pit and fissure sealants and preventive resin restoration, fluoride varnish/gel, and space maintainer interventions during 2020 compared to 2019 [26].

A significant difference is seen in deciduous tooth extraction between the year before the pandemic and the consequent two years. Although we saw a decrease in this therapeutic intervention between the year before and the first year of the pandemic, if we look at the percentage/prevalence of individual interventions carried out in relation to the total number of interventions by each examined year, we can see that the extractions were more prevalent in the first and second years of COVID-19. A similar situation is seen in a study from Jeddah City, Saudi Arabia, where the most common procedure performed during the COVID-19 period compared to the previous year was extraction, with 406 (58.10%) in the year of the pandemic and 619 (53.80%) cases in the previous year [11].

As previously mentioned, the most prevalent procedures in the first year of COVID-19 were restorative procedures, especially glass ionomer filling. Glass ionomer filling is the most used material in paediatric restorative dentistry. It is hydrophilic and tolerates a moist environment, so it is used in atraumatic restorative treatment (ART). For this treatment, no high-speed handpiece is needed, and no aerosols are generated. The use of dental handpieces can increase the risk of exposure and transmission of COVID-19 between patients and healthcare workers [27,28]. The study by Alamoudi et al. also found that ART was used more frequently in its sample of paediatric patients compared to the same period in the pre-COVID-19 year [11]. Although our result does not refer to ART itself, this kind of treatment is frequently used in our clinic.

Regarding endodontic procedures from our study, these were less prevalent in the three study periods. Nevertheless, a statistically significant difference was found in relation to the examined periods. In addition, Al Hayyan et al. reported a similar significant increase in restorative and endodontic procedures during the period from July 2020 to the end of the study in 2021 compared to the pre-COVID period ending in April 2019 [7].

There are certain limitations of this study. The study analysed only the situation in our clinic, which might not be applicable to some other countries or areas. Another limitation of this study is that it was conducted retrospectively, and the cross-sectional survey could not be used to determine causalities. Since we do not have information about the extent of antibiotic prescriptions before and during the COVID-19 period, we wonder if this could be a possible confounder in regard to patient visits. Future investigation may clarify that matter.

Despite these limitations, the study has some strengths, including the fact that the study followed the population not only into one year of the pandemic (2020) but into the second year (2021), which may show a clearer picture of the longer-term impacts of COVID-19-related changes on the paediatric dentistry practice. Furthermore, sharing knowledge and learning from past experiences in a different setting would enable better organization of oral health services. With current experience, we suggest that dental systems in clinics should rely more on teledentistry and digital systems in handling patients’ needs so we could be better prepared in the future. In countries such as Bosnia and Herzegovina, prevention programs in dentistry should be better organized and implemented as this was a major issue during the periods before and, also, during COVID-19.

## 5. Conclusions

The results of this study show a significant decrease in the types of procedures performed, especially preventive and prophylactic dental treatment, in the first year of the pandemic. Although the number of dental treatments in the second year in the clinic nearly returned to pre-pandemic levels, preventive and restorative interventions are the most appropriate strategy to improve the oral health of children after the COVID-19 pandemic.

## Figures and Tables

**Table 1:** Newly registered patients attending the Department of Paediatric Dentistry of the Dental Clinic in Banja Luka and data on number of dental treatments during the three time periods considered.

	Before Pandemic *n*	First Year *n*	Second Year *n*	*p*-Value (χ^2^)
New registered patients	242	181	337	<0.01 * (73.1)
Dental treatments	2757	1652	2585	0.7 (196.3)

* *p* < 0.05 statistical significance.

**Table 2:** Comparison and correlation among the treatment procedures of paediatric patients before and during the first year of the COVID-19 pandemic.

Dental Treatment Procedures	Before Pandemic *n* (% **)	First Year *n* (% **)	χ^2^	*p*-Value
Oral hygiene training and patient motivation	327 (11.9%)	186 (11.3%)	0.2	0.6
Fluoride varnish	580 (21.1%)	190 (11.5%)	468.1	<0.01 *
Fissure sealant	186 (6.7%)	93 (5.6%)	19.2	0.2
Dental plaque removal	351 (12.7%)	69 (4.1%)	74.0	<0.01 *
Deciduous tooth extraction	346 (12.5%)	262 (15.8%)	71.6	<0.01 *
Glass ionomer filling	396 (14.4%)	354 (21.4%)	255.4	<0.01 *
Composite filling on anterior teeth	92 (3.3%)	46 (2.8%)	0.9	0.3
Composite filling on posterior teeth	257 (9.3%)	214 (12.9%)	11.4	<0.01 *
Temporary filling	100 (3.6%)	99 (6.0%)	121.8	<0.01 *
Temporary endodontic procedures	81 (2.9%)	130 (7.9%)	495.3	<0.01 *
Root canal filling	41 (1.5%)	9 (0.5%)	8.0	<0.01 *
Pearson’s Correlation	r2 = 0.6; *p* = 0.03 *			

* *p* < 0.05 statistical significance, ** %-prevalence of single procedure regarding the number of dental treatments by each year.

**Table 3:** Comparison and correlation among the treatment procedures of paediatric patients before and during the second year of the COVID-19 pandemic.

Dental Treatment Procedures	Before Pandemic *n* (% **)	Second Year *n* (% **)	χ^2^	*p*-Value
Oral hygiene training and patient motivation	327 (11.8%)	362 (14.0%)	4.2	0.04 *
Fluoride varnish	580 (21.1%)	403 (15.6%)	182.1	<0.01 *
Fissure sealant	186 (6.7%)	186 (7.2%)	0.4	0.5
Dental plaque removal	351 (12.7%)	115 (4.1%)	968.7	<0.01 *
Deciduous tooth extraction	346 (12.5%)	435 (16.8%)	145.6	<0.01 *
Glass ionomer filling	396 (14.3%)	510 (19.7%)	19.3	<0.01 *
Composite filling on anterior teeth	92 (3.3%)	54 (2.1%)	74.0	<0.01 *
Composite filling on posterior teeth	257 (9.3%)	284 (10.9%)	3.3	0.1
Temporary filling	100 (3.6%)	76 (2.9%)	18.5	0.2
Temporary endodontic procedures	81 (2.9%)	148 (5.7%)	231.7	<0.01 *
Root canal filling	41 (1.4%)	12 (0.4%)	13.9	<0.01 *
Pearson’s Correlation	r2 = 0.8; *p* < 0.01 *			

* *p* < 0.05 statistical significance; ** %-prevalence of single procedure regarding the number of dental treatments by year.

**Table 4:** Comparison and correlation among the treatment procedures of paediatric patients during the first and second years of the COVID-19 pandemic.

Dental Treatment Procedures	First Year *n* (% **)	Second Year *n* (% **)	χ^2^	*p*-Value
Oral hygiene training and patient motivation	186 (11.3%)	362 (14.0%)	5.2	0.02 *
Fluoride varnish	190 (11.5%)	403 (15.6%)	10.6	<0.01 *
Fissure sealant	93 (5.6%)	186 (7.2%)	35.3	0.06
Dental plaque removal	69 (4.1%)	115 (4.1%)	0.2	0.6
Deciduous tooth extraction	262 (15.8%)	435 (16.8%)	0.5	0.4
Glass ionomer filling	354 (21.4%)	510 (19.7%)	1.2	0.2
Composite filling on anterior teeth	46 (2.8%)	54 (2.1%)	20.1	0.1
Composite filling on posterior teeth	214 (12.95%)	284 (10.9%)	2.9	0.08
Temporary filling	99 (6.0%)	76 (2.9%)	21.7	<0.01 *
Temporary endodontic procedures	130 (7.9%)	148 (5.7%)	65.9	0.01 *
Root canal filling	9 (0.5%)	12 (0.5%)	0.13	0.7
Pearson’s Correlation	R = 0.9; *p* < 0.01 *			

* *p* < 0.05 statistical significance; ** %-prevalence of single procedure regarding the number of dental treatments by year.

## Data Availability

The data that support the findings of this study are available upon reasonable request from the corresponding author.

## Abbreviations

SARS-CoV-2	Severe acute respiratory syndrome coronavirus 2
COVID-19	Coronavirus disease 19
WHO	World Health Organization
ART	Atraumatic Restorative Treatment

## References

[1] Tonkaboni A., Amirzade Iranaq M.H., Ziaei H., Ather A. (2021). Impact of COVID-19 on dentistry. Adv. Exp. Med. Biol..

[2] Mallineni S.K., Innes N.P., Raggio D.P., Araujo M.P., Robertson M.D., Jayaraman J. (2020). Coronavirus disease (COVID-19): Characteristics in children and considerations for dentists providing their care. Int. J. Paediatr. Dent..

[3] Ferrazzano G.F., Ingenito A., Cantile T. (2020). COVID-19 disease in children: What dentists should know and do to prevent viral spread. The Italian point of view. Int. J. Environ. Res. Public Health.

[4] World Health Organization Considerations for the Provision of Essential Oral Health Services in the Context of COVID-19: Interim Guidance, 3 August 2020. https://www.who.int/publications/i/item/who-2019-nCoV-oral-health-2020.1.

[5] Cucinotta D., Vanelli M. (2020). WHO declares COVID-19 a pandemic. Acta Biomed.

[6] Amato A., Caggiano M., Amato M., Moccia G., Capunzo M., De Caro F. (2020). Infection control in dental practice during the COVID 19 pandemic. Int. J. Environ. Res. Public Health.

[7] AlHayyan W.A., AlShammari K., AlAjmi F., Pani S.C. (2022). The Impact of COVID-19 on Dental Treatment in Kuwait—A Retrospective Analysis from the Nation’s Largest Hospital. Int. J. Environ. Res. Public Health.

[8] Jayaraman J., Dhar V., Moorani Z., Donly K., Tinanoff N., Mitchell S., Wright T. (2020). Impact of COVID-19 on pediatric dental practice in the United States. Pediatr. Dent..

[9] Ahmadi H., Ebrahimi A., Ghorbani F. (2020). The impact of COVID-19 pandemic on dental practice in Iran: A questionnaire-based report. BMC Oral Health.

[10] Yang J., Yang G., Jin R., Song G., Yuan G. (2022). Changes in paediatric dental clinic after reopening during COVID-19 pandemic in Wuhan: A retrospective study. BMJ Open.

[11] Alamoudi R.A., Basudan S., Mahboub M., Baghlaf K. (2022). Impact of COVID-19 Pandemic on Dental Treatment in Children: A Retrospective Cross-Sectional Analysis in Jeddah City. Clin. Cosmet. Investig. Dent..

[12] Cagetti M.G., Balian A., Camoni N., Campus G. (2021). Influence of the COVID-19 pandemic on dental emergency admissions in an urgent dental care service in North Italy. Int. J. Environ. Res. Public Health.

[13] Nijakowski K., Cieślik K., Łaganowski K., Gruszczyński D., Surdacka A. (2021). The Impact of the COVID-19 Pandemic on the Spectrum of Performed Dental Procedures. Int. J. Environ. Res. Public Health.

[14] Gómez-Costa D., Ramírez J.M., García Guerrero I., Giovannini G., Rojo R., Gómez-de Diego R. (2022). A retrospective study on the effect of the COVID-19 pandemic on dental treatments in adults. BMC Oral Health.

[15] Live COVID-19 Vaccination Tracker. https://covidvax.live/location/bih.

[16] Casamassimo P.S., Townsend J.A., Litch C.S. (2020). Pediatric dentistry during and after COVID-19. Pediatr. Dent..

[17] Ilyas N., Agel M., Mitchell J., Sood S. (2020). COVID-19 pandemic: The first wave—An audit and guidance for paediatric dentistry. Br. Dent. J..

[18] Paglia L. (2020). COVID-19 and paediatric dentistry after the lockdown. Eur. J. Paediatr. Dent..

[19] Vuković A., Mandić-Rajčević S., Sava-Rosianu R., Betancourt D.M., Xhajanka E., Hysenaj N., Campus G. (2021). Pediatric Dentists’ Service Provisions in South-East Europe during the First Wave of COVID-19 Epidemic: Lessons Learned about Preventive Measures and Personal Protective Equipment Use. Int. J. Environ. Res. Public Health.

[20] Dolic O., Vojinovic J., Djukanovic D., Cupic S., Sukara S., Obradovic M., Kojic Z., Trtic N. (2010). Caries prevalence in the primary and permanent dentition of rural and urban children in the municipality of Banja Luka, Bosnia and Herzegovina. Oral Health Dent. Manag..

[21] Obradović M., Dolić O., Sukara S., Knežević N., Kojić Ž. (2020). Identifying risk factors of severe early childhood caries in infants from Bosnia and Herzegovina. Cent. Eur. J. Public Health.

[22] Eggmann F., Haschemi A.A., Doukoudis D., Filippi A., Verna C., Walter C., Weiger R., Zitzmann N.U., Bornstein M.M. (2021). Impact of the COVID-19 pandemic on urgent dental care delivery in a Swiss university center for dental medicine. Clin. Oral Investig..

[23] OECD iLibrary Impact of COVID-19 in Bosnia and Herzegovina. https://www.oecd-ilibrary.org/sites/f0011603-en/index.html?itemId=/content/component/f0011603-en.

[24] Ibrahim M.S., Alibrahim H., Al Madani A., Alamri A., Bamashmous M., Tounsi A. (2021). Fear factor in seeking dental care among Saudis during COVID-19 pandemic. Int. J. Environ. Res. Public Health.

[25] Elalouf A., Moran R., Yaron B., Oman M. (2022). Pediatric Dental Emergency Visits and Treatment during Lockdown in the COVID-19 Pandemic: A Retrospective Study. Int. J. Environ. Res. Public Health.

[26] Samuel S.R., Mathew M.G., Suresh S.G., Varma S.R., Elsubeihi E.S., Arshad F., Elkareimi Y., Elsahn N.A., Khalil E. (2021). Pediatric dental emergency management and parental treatment preferences during COVID-19 pandemic as compared to 2019. Saudi J. Biol. Sci..

[27] Abramovitz I., Palmon A., Levy D., Karabucak B., Kot-Limon N., Shay B., Kolokythas A., Almoznino G. (2020). Dental care during the coronavirus disease 2019 (COVID-19) outbreak: Operatory considerations and clinical aspects. Quintessence Int..

[28] Cirillo N. (2020). COVID-19 outbreak: Succinct advice for dentists and oral healthcare professionals. Clin. Oral Investig..

